# Draft Genome Sequence of an Environmental *trh*^+^ *Vibrio parahaemolyticus* K23 Strain Isolated from Kerala, India

**DOI:** 10.1128/genomeA.00282-16

**Published:** 2016-04-14

**Authors:** Divya Meparambu Prabhakaran, Goutam Chowdhury, Gururaja Perumal Pazhani, Thandavarayan Ramamurthy, Sabu Thomas

**Affiliations:** a Cholera and Biofilm Research Laboratory, Rajiv Gandhi Centre for Biotechnology, Trivandrum, Kerala, India; b National Institute of Cholera and Enteric Diseases, Kolkata, India; c Translational Health Science and Technology Institute, Faridabad, Haryana, India

## Abstract

*Vibrio parahaemolyticus* is the leading cause of seafood-related gastroenteritis. Here, we report the draft genome sequence of a *trh*^+^ strain, *V. parahaemolyticus* K23, isolated from seafood. The sequence will be useful for comparative analysis between environmental and clinical isolates of *V. parahaemolyticus*.

## GENOME ANNOUNCEMENT

*Vibrio parahaemolyticus*, a Gram-negative halophilic bacterium inhabiting estuarine and marine environments, is associated with severe gastroenteritis following consumption of raw or undercooked seafood. Pathogenicity of this organism has been classically associated with thermostable direct hemolysin (TDH), TDH-related hemolysin (TRH) adhesins, type III (T3SS) and type VI secretion systems (T6SS) (1). Generally, environmental *V. parahaemolyticus* is considered nonpathogenic (2). However, recent reports have identified one or more of these virulence-encoding genes in strains isolated from the environment and seafood (3, 4). We isolated *V. parahaemolyticus*, an O4:K36 serovar having the TRH gene and *tox*RS/*new* sequence, from mussel collected from the Ashtamudi estuarine system of Kollam, Kerala, India, in 2013. The isolate K23 has been deposited in the Gastrointestinal Tract Pathogens Repository (GTPR) maintained by the National Institute of Cholera and Enteric Diseases, Kolkata, India, under GTPR number 1398. This isolate was found to harbor genes encoding for T3SS2β apparatus proteins (*vscC2*, *vscS2*, *vopB2*) and effectors VopC, VopP, and VopL, a virulence profile predicted in clinical strains (unpublished data)*.* We therefore sequenced *V. parahaemolyticus* K23 to understand the overall pathogenic potential of this toxigenic environmental isolate and also to identify differences in the two T3SS phylotypes (α and β), if any.

The isolate K23 was grown at 37°C overnight in Luria-Bertani broth supplemented with 3% NaCl (Difco Laboratories, Detroit, MI, USA), and the genomic DNA was extracted using the GenElute bacterial genomic DNA kit (Sigma-Aldrich, St. Louis, MO, USA) according to the manufacturer’s instructions. One microgram of gDNA was used for library preparation using the TruSeq DNA library generation kit (Illumina, San Diego, CA, USA). Paired-end sequencing was performed on the Illumina MiSeq platform generating 1,867,264 reads (2 × 150-bp) at 140× coverage. Reads were trimmed and assembled *de novo* into 74 contigs (*N*_50_, 410,730 bp) using SPAdes version 3.0.0 (5). The largest contig length was 856,967 bp. The sequences were annotated using the NCBI Prokaryotic Genome Automatic Annotation Pipeline (6) and analyzed with the Rapid Annotations using Subsystems Technology (RAST) server (7). The genome has a size of 5,090,551 bp and a G+C content of 45.3%, which is similar to that of other *V. parahaemolyticus* strains. The annotation process identified 4,481 protein coding sequences, 56 pseudogenes, 16 rRNAs, 102 tRNAs, and 1 ncRNA; 547 subsystems were identified by RAST. Genes encoding urease cluster, TRH, T3SS apparatus proteins and effectors, and T6SS were also identified. A detailed comparison of *V. parahaemolyticus* K23 with other sequenced *V. parahaemolyticus* genomes will be discussed elsewhere.

### Nucleotide sequence accession numbers.

This whole-genome shotgun project has been deposited at DDBJ/EMBL/GenBank under the accession number LQGU00000000. The version described in this paper is the first version, LQGU01000000.
